# Promising photocatalytic and antimicrobial activity of novel capsaicin coated cobalt ferrite nanocatalyst

**DOI:** 10.1038/s41598-023-32323-y

**Published:** 2023-04-01

**Authors:** Ahmed M. El-Khawaga, Mohamed A. Elsayed, Yosri A. Fahim, Rasha E. Shalaby

**Affiliations:** 1 Department of Basic Medical Sciences, Faculty of Medicine, Galala University, Galala City, 43511 Suez Egypt; 2 Chemical Engineering Department, Military Technical College (MTC), Egyptian Armed Forces, Cairo, Egypt; 3 Department of Microbiology and Immunology, Faculty of Medicine, Tanta University, Tanta, Egypt

**Keywords:** Environmental sciences, Nanoscience and technology

## Abstract

In this study, CoFe_2_O_4_ nanoparticles were prepared by the co-precipitation method then surface modified with Capsaicin (*Capsicum annuum ssp.*). The virgin CoFe_2_O_4_ NPs and Capsaicin-coated CoFe_2_O_4_ NPs (CPCF NPs) were characterized by XRD, FTIR, SEM, and TEM. The antimicrobial potential and photocatalytic degradation efficiencies of the prepared samples via Fuchsine basic (FB) were investigated. The results revealed that CoFe_2_O_4_ NPs have spherical shapes and their diameter varied from 18.0 to 30.0 nm with an average particle size of 25.0 nm. Antimicrobial activity was tested on Gram-positive (*S. aureus*ATCC 52923) and Gram-negative (*E. coli* ATCC 52922) by disk diffusion and broth dilution methods to determine the zone of inhibition (ZOI) and minimum inhibitory concentration (MIC), respectively. UV-assisted photocatalytic degradation of FB was examined. Various parameters affecting the photocatalytic efficiency such as pH, initial concentration of FB, and dose of nanocatalyst were studied. The in-vitro ZOI and MIC results verified that CPCF NPs were more active upon Gram-Positive *S. aureus* ATCC 52923 (23.0 mm ZOI and 0.625 μg/ml MIC) than Gram-Negative *E. coli* ATCC 52922 (17.0 mm ZOI and 1.250 μg/ml MIC). Results obtained from the photocatalytic activity indicated that the maximum FB removal achieving 94.6% in equilibrium was observed using 20.0 mg of CPCF NPS at pH 9.0. The synthesized CPCF NPs were effective in the removal of FB and also as potent antimicrobial agent against both Gram-positive and Gram-negative bacteria with potential medical and environmental applications.

## Introduction

Nanotechnology, specifically objects smaller than 100 nm, is the science and technology of precisely changing the molecular structure of matter. The last ten years have seen significant advancements in catalysis known as "nanocatalysis" and the emergence of a new technological revolution. A popular area of research is nanocatalysis, which involves using nanoparticles as catalysts in a number of catalysis processes^1^. Due to the fact that when a material's size is reduced to the nanoscale, the surface area is greatly increased and the substance can be evenly disseminated in solution to produce a homogenous emulsion, nanocatalysts are an appealing replacement for conventional catalysts^2^. By adjusting the chemical and physical characteristics of nanocatalysts, such as their size, shape, composition, and morphology, one can significantly boost their catalytic activity, selectivity, and stability^3^. Researchers have received a great attention to the removing of cationic dyes from water because of the harmful effects they might cause in ecosystems^4^. The presence of these contaminants in water sources decreases the quality of the water. The global water situation is deteriorating on every country. Wastewater treatment appears to be an appropriate solution for this issue^5^. As a result, nanocatalysts play an important role in photocatalytic degradation of dyes, but isolation and recovering them from the reaction media is typically a difficult, time-consuming, and expensive process due to their extremely small size^6^. Magnetic nanocatalysts can be quickly extracted from the reaction medium using an external magnet, without a need for more filtration, centrifugation, or other time-consuming methods^7^. Magnetic nanoparticles (MNPs) have a number of superior properties, including high surface area to bulk ratios, low toxicity, high activity, thermal stability, surface modification, and dispersibility^7–10^. As a result, they are more appropriate catalysts or supports and more sustainable than ordinary samples^11^. Due to their strong anisotropy, high coercivity, moderate saturation magnetization, good mechanical and excellent chemical stabilities at higher temperature, which are significantly different from their bulk counterparts, Cobalt ferrite nanoparticles (CoFe_2_O_4_ NPs) have drawn significant attention among these magnetic nanoparticles^12,13^. Cobalt ferrites are employed often in sensors, recording devices, magnetic cards, solar cells, magnetic drug delivery, healthcare, catalysis, and biotechnology because of these properties^14^. CoFe_2_O_4_ nanoparticles have been synthesized using a variety of preparation methods, including microemulsion^15^, sol–gel techniques^16^, hydrothermal synthesis^17^, solvothermal method^18^, co-precipitation^19^ and green synthesis method by using Plant extract, Bacteria, Fungi and algea as biological agents for generation of nanomaterials^20^. Co-precipitation technique is one of these techniques, and it is simple and inexpensive to use to make cobalt ferrite nanoparticles. Co-precipitation has a variety of advantages including being rapid, simple, versatile, and inexpensive^21^. Unfortunately, because of their high surface energy and strong magnetic dipole interactions, cobalt ferrites are extremely susceptible to agglomeration^22^. The best way to date has been found to be the modification of ferrite nanoparticles using appropriate stabilising coating materials^23^. Use of plant extracts for the synthesis and coating of nanoparticles has many advantages, such as being cost-effective, eco-friendly, and also taking place of the process in one setup; also, nanoparticles act as carrier in the transfer of materials into cells^24^. Medicinal plants have therapeutic properties due to the presence of various complex chemical substances of different compositions, which are found as plant metabolites in certain parts of the plants^25^. Capsaicin, a potent alkaloid, has the ability to stabilise the surface of cobalt ferrite.

**Figure 1:**
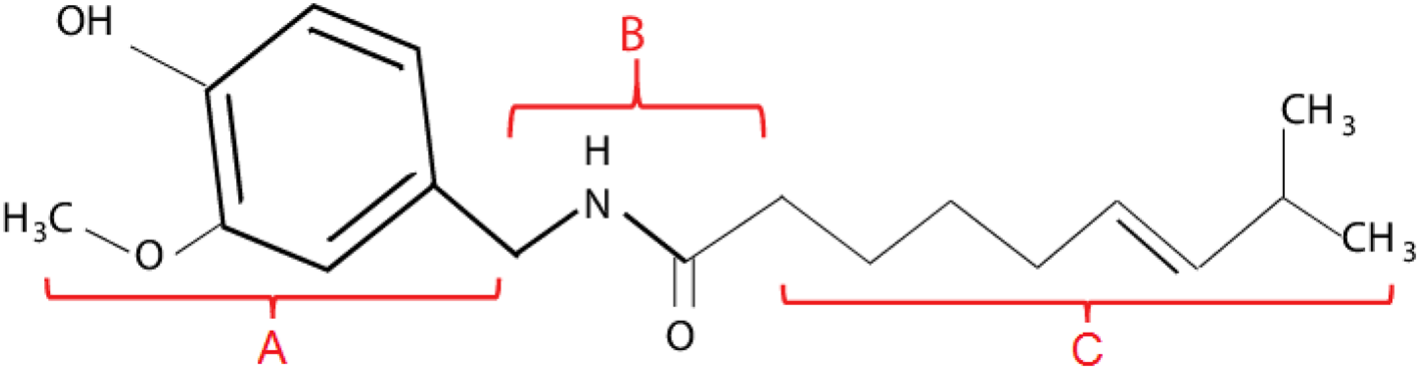
Chemical structure of capsaicin.

The structure of the capsaicin molecule can be divided into three distinct regions, [A] as a vanillyl group, [B] as an amide bond, and [C] as a fatty acid chain (Fig. 1)^26^. Table 1 shows the chemical formula and properties of Capsaicin^27,28^**.** In addition to its many physiological and pharmacological advantages (pain relief, cancer prevention, favourable cardiovascular, and gastrointestinal effects), capsaicin has most recently drawn a lot of interest due to its antibacterial and anti-virulence potential. A bactericidal activity has been demonstrated against Helicobacter pylori, and Pseudomonas aeruginosa^27^.

**Table 1:** The chemical formula and properties of the Capsaicin molecule.

	Properties
Chemical formula	C_18_H_27_NO_3_
Molar mass	305.418 g mol^−1^
Appearance	Crystalline white powder
Odor	Highly volatile and pungent
Melting point	62–65 °C (144–149 °F; 335–338 K)
Boiling point	210–220 °C (410–428 °F; 483–493 K) 0.01 Torr
Solubility in water	0.0013 g/100 mL
Vapor pressure	1.32 × 10^−8^ mm Hg at 25 °C
UV–Vis (λ_max_)	280 nm

Finally, this paper presents an investigation of Capsaicin—coated CoFe_2_O_4_ (CPCF NPs) synthesis in nanometric sizes by co-precipitation method and the evaluation of the antimicrobial activity and photocatalytic potential of these structures for the degradation of Fuchsine basic (FB).

## Materials and methods

### Materials

Ferric nitrate Fe (NO_3_)_3_·6H_2_O), Cobalt nitrate (Co (NO_3_)_2_· 4 H_2_O), Sodium hydroxide, and ethanol 96% (v/v) were obtained from (Merck, India). All the chemicals were reagent grade and used without further purification. Water used throughout the experiment was ultrapure milli-Q water.

### Capsaicin extraction from hot pepper (*Capsicum annuum *ss*p)*

Several organic solvents can be to extract capsaicin from hot peppers, but only ethanol is appropriate for producing pharmaceutical-grade material^29,30^. The dried and crushed Capsicum annuum ssp. was kept in desiccators and used for obtaining the capsaicin^31^. Extraction was performed using (0.1–0.5 g of powdered plant material was taken for extraction) with 96% (v/v) ethanol, in a water bath at 40 °C for 5 h. Then, water vacuum filtration for obtaining an ethanol extract of capsaicin^31^**.**

### Synthesis of cobalt ferrite nanoparticles

**Figure 2:**
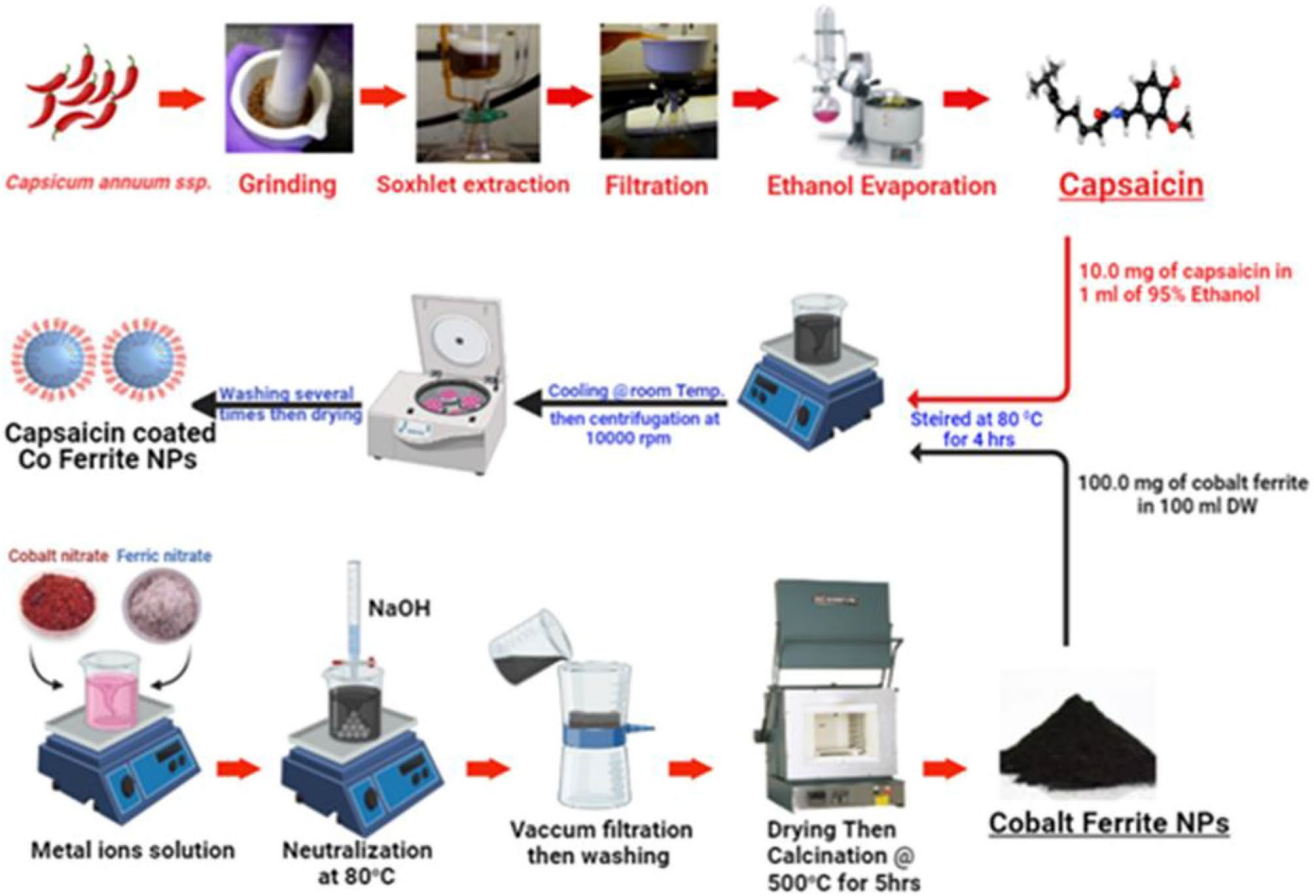
The systematic representation of the preparation of (CPCF) NPs.

Cobalt ferrite nanoparticles (CF NPs) are synthesized via coprecipitation method as reported previously by Vinosha et al.^32^**.** Initially, Cobalt nitrate (0.1 M) and ferric Nitrate (0.2 M) were dissolved separately in 100 mL of distilled water and stirred to obtain a clear solution. Then sodium hydroxide (1 M) was added dropwise to achieve pH 9 under continuous stirring. The obtained precipitate was stirred at 80 °C for 3 h. As a result, the brown precipitate was washed thrice with double distilled water and twice with ethanol. The obtained product was dried at 80 °C for 24 h in an oven to obtain the final product of CoFe_2_O_4_ nanoparticles^33^. The sample thus obtained was characterized. Figure 2 illusterate The schematic representation of the capsaicin coated Cobalt ferrite magnetic nanoparticles preparation.

### Preparation of capsaicin coated cobalt ferrite (CPCF) magnetic nanoparticles

Capsaicin coated with cobalt ferrite nanoparticles was synthesized by adding an ethanolic capsaicin solution to CF NPs. Firstly, 10 mg of capsaicin was added to ethanolic solution (1 ml of 95% ethanol) then mixed with 100 mg of CF NPs. The resulting ethanolic mixture was agitated and finnaly placed in a rotatory evaporator until all of the ethanol had evaporated^34^.

### Characterization methods

The surface functionality of the synthesized nanoparticles were confirmed by FTIR spectra (JASCO FT-IR 3600 Infra-Red spectrometer). All samples were prepared in KBr in the range of 400–4000 cm^−1^. The phase analysis of the synthesized nano-powder was performed on an X’pert Pro Phillips X-ray diffractometer. High-resolution transmission electron microscopy (HRTEM, JEOL 3010, Japan) operated at 300 kV was used to examine the size and morphology of synthesised nanoparticles. The surface structure of the synthesized magnetic nanoparticles were characterized by scanning electron microscopy (SEM) ZEISS, EVO-MA10, Germany. The coating of CoFe_2_O_4_ NPs with Capsaicin was determined using UV–Vis spectroscopy on an Agilent Cary 60 UV–Vis. spectrophotometer.

### Antimicrobial activity and minimal inhibitory concentration (MIC)

The synthesized (CPCF) nanocomposite, CoFe_2_O_4_ NPs (20.0 μg/ml) were evaluated for their antimicrobial activity by agar-disc distribution method^35^, towards Bacterial strains from American Type Culture Collections (ATCC) strains, namely, Gram-negative (*Escherichia coli* ATCC 25922) and Gram-positive (*Staphylococcus aureus* ATCC 25923) bacterial strains. Conventional antibiotic discs (E) Erythromycin; 20 μg/ml; 6.0 mm diameter), was chosen to determine the performance of the tested magnetic nanocomposite. The minimum inhibitory concentrations (MIC) of the tested samples which have the highest antimicrobial activity was determined by The serial dilutions method of Luria–Bertani (LB) medium^36^**.** For these determinations, The synthesized (CPCF) nanocomposite, and CoFe_2_O_4_ NPs (beginning with concentration = 20.0 μg/ml) were applied. The medium broth act as a negative control and the medium broth inoculated with the examined microbes act as a positive control such. MIC was determined next 24 h. of incubation at 36.0 ± 1.0 °C^37^. The resultss are statistically treated by using ONE WAY ANOVA, Duncan's multiple series, and the least significant difference (LSD) that are determined by specific software (SPSS version 15)^38^.

### Photocatalytic degradation of fuchsine basic (FB) using nanocomposite

The CPCF nanocomposite (10 mg) obtained as in Section "Preparation of capsaicin coated cobalt ferrite (CPCF) magnetic nanoparticles" was added to 50 ml of an aqueous solution of FB with initial concentration C_0_ = 10 mg l^−1^, under constant stirring at ambient temperature (25 °C) for 30 min in the dark, until adsorption–desorption equilibrium was attained between FB and the prepared photocatalyst (nanocomposite). After that, a UV lamp was used as a source of UV light to irradiate the solution containing the nanocatalyst and FB. At constant time intervals of irradiation, 1 ml of sample was draw outusing a syringe equipped with a filter (2.5 mm pore size). The FB degradation rate was calculated by determining the variation in FB concentration versus irradiation time using a UV–vis spectrophotometer (Agilent Technologies Cary 60 UV–vis) at λ_max_ = 546 nm. DI water was used as a reference blank^39^. The percentage degradation was calculated using the following formula^40^:

1 $$\% {\text{ Degradation }} = \, \left( {{\text{C}}_{0} - {\text{ C}}_{t} } \right)/{\text{C}}_{0} \times \, 100$$

## Results and discussion

### Characterization of the prepared CoFe_2_O_4_ NPs and CPCF NPs

The CoFe_2_O_4_ NPs were prepared by a chemical co-precipitation method with minor modifications^32,33,41^. The reaction was carried out as in the equations below:

2 $$2{\text{Fe}}^{3 + } + {\text{ Co}}^{2 + } \to {\text{Fe}}_{2} {\text{Co }}\left( {{\text{OH}}} \right)_{8}$$

3 $${\text{Fe}}_{2} {\text{Co }}\left( {{\text{OH}}} \right)_{8} \to {\text{CoFe}}_{2} {\text{O}}_{4} + \, 4{\text{H}}_{2} {\text{O}}$$

#### FTIR spectra analysis

**Figure 3:**
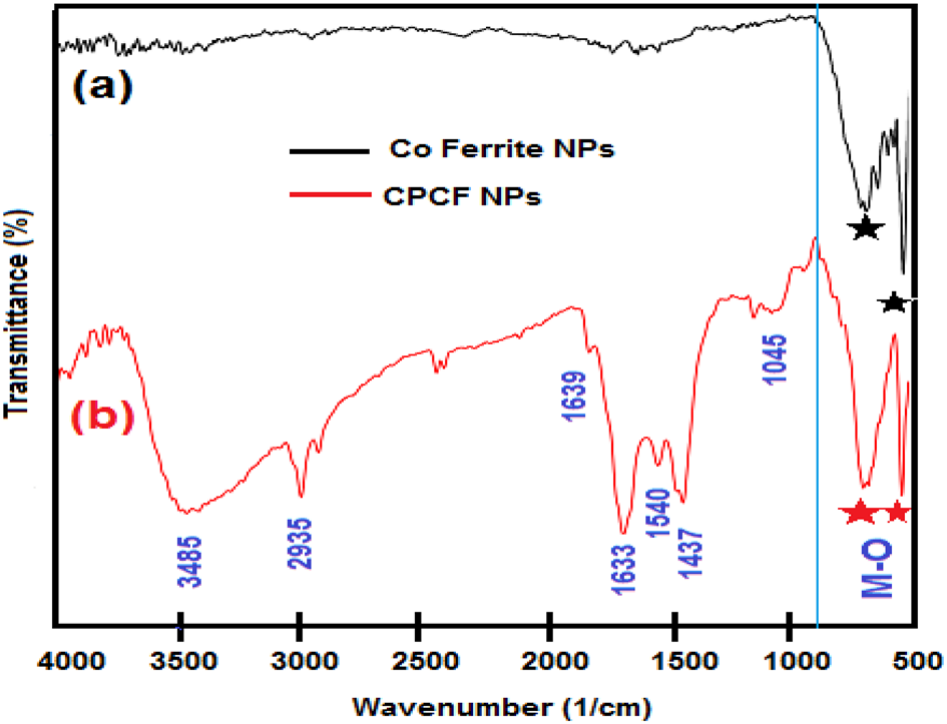
FTIR Spectrum of (**a**) Co Ferrite NPs (**b**) Capsaicin coated Co ferrite (CFCP) NPs.

FTIR analysis was used to confirm the functional groups on the surface of the synthesized Magnetic NPs. The spectra of pure Co Fe_2_O_4_ NPs and CPCF NPs were represented in Fig. 3. The stretching vibration mode associated with the Fe–O bond in the crystalline lattice of CoFe_2_O_4_ NPs was attributed to the presence of strong peaks of CoFe_2_O_4_ NPs and CPCF NPs at 658 cm^−1^. Furthermore, the band at 515 cm^−1^ was attributed to the metal oxide bond (Co–O) in the nanoparticles^42^. The IR spectra for the (CPCF) nanocomposite show peak at 3485 cm^−1^ which is attributed to the O–H stretching vibrations while at 2935 cm^−1^ is attributed to the symmetric C–H stretching vibrations. The peaks at 1045 cm^−1^may are attributed to C–O–C stretching of ether. The peaks at 1633 and 1639 cm^−1^ may be attributed to (C=O) stretching vibrations. Also, the presence of peaks between (1437–1540 cm^−1^) may be due to (C–C) stretching vibration in the aromatic rings^43^.

#### XRD analysis

**Figure 4:**
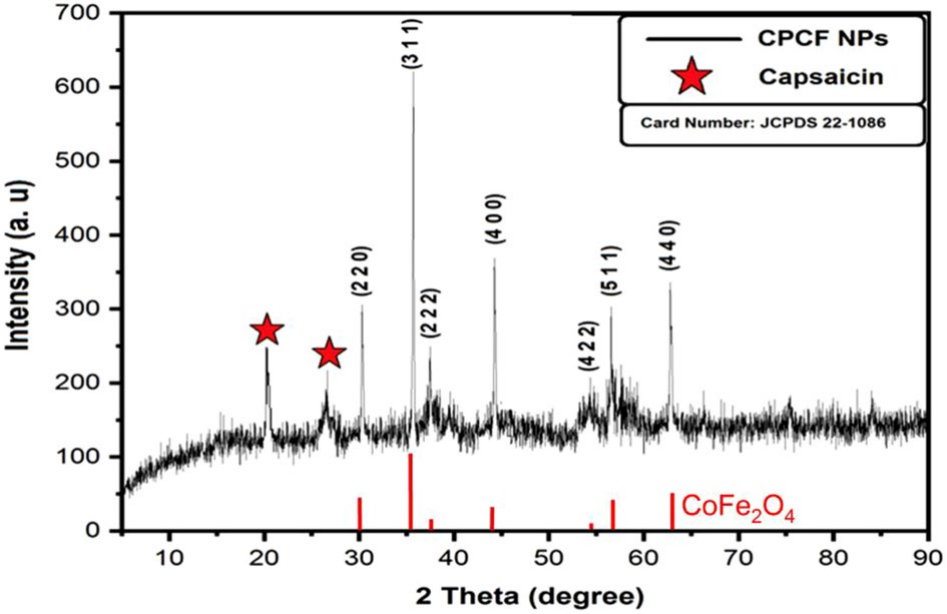
XRD pattern of capsaicin coated Co ferrite nanopowder.

The XRD analysis of Capsaicin coated Co Ferrite (CPCF) nanopowder which was treated and annealed up to 150 °C for 24 h was presented in (Fig. 4). The obseved diffraction peaks of synthesized CoFe_2_O_4_ NPs were matched well with the diffraction standard (JCPDS 22-1086), and give persuasive evidence for the generation of the cubic spinel structure of cobalt ferrite Nanoparticles. The observed diffraction peaks at 2θ = 30.0°, 35.9°, 37.2, 44.0°, 54.0°, 57.0° and 63.0° were corresponded to (220), (311), (222), (400), (422), (511), and (440) planes of cubic spinel structure of CoFe_2_O_4_ NPs respectively. The average crystal size was reported to be 18.35 nm, and the lattice parameter was determined to be 8.439 A. The XRD pattern indicates that the synthesized sample is in the nanoscale range. In addition, the diffraction peaks at 20.25 ^o^ and 28.0° corresponded to Capsaicin^44^, confirming the loading of capsaicin with the synthesized CoFe_2_O_4_ NPs. The crystallite size of the synthesized NPs was caculated using Scherrer’s Equation^45^:

4 $${\text{D}} = 0.9\lambda /\beta \cos \theta$$

where, D is the crystallite size, λ is the X-ray wavelength used, β is the full width at half maximum (FWHM) and θ is the diffraction angle. The crystallite size was found to be 18.35 nm at the strongest peak at (311) plane. Particle size is a crucial factor affecting the performance of nano-photocatalytic materials. The size and shape of the catalyst influence its surface structure and then resulting in various catalytic performance^46^. CPCF nanoparticles have a large surface area and a broadened band gap, furthermore, they contain more active sites and display improved photocatalytic activity.

#### Scanning electron microscopy (SEM) and transmission electron microscopy (TEM) analysis

**Figure 5:**
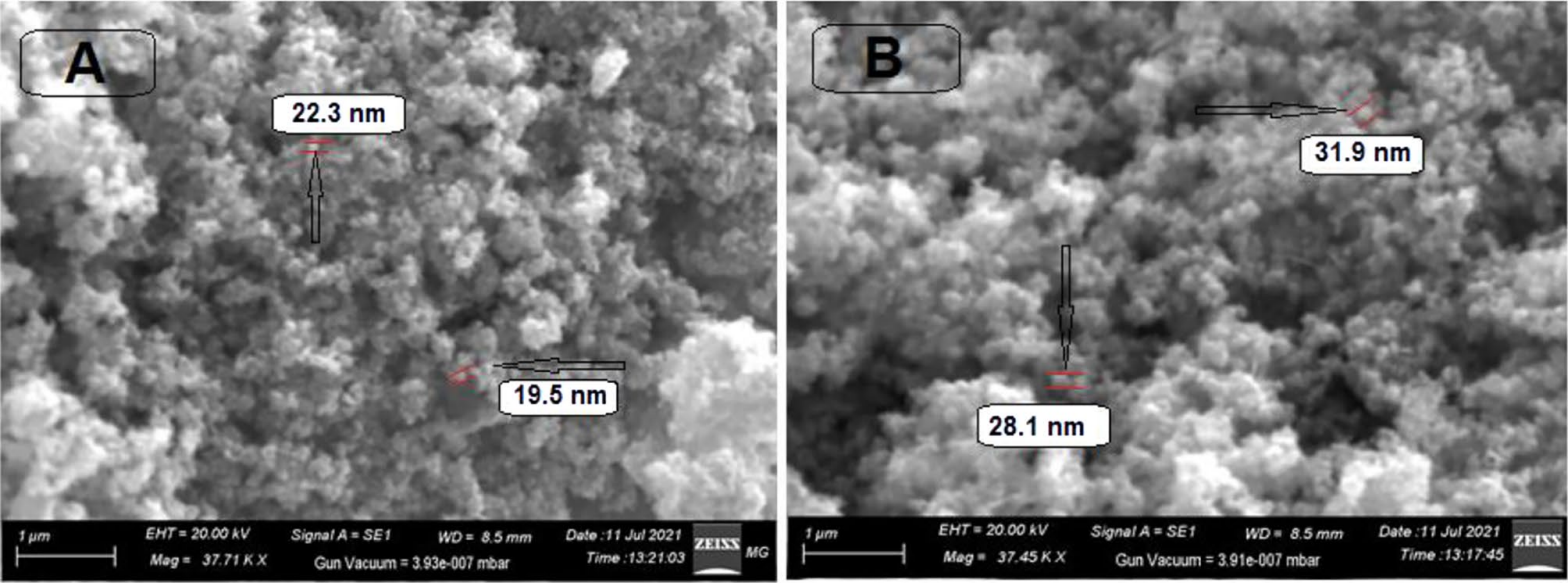
SEM images of (**A**) Nacked Co Ferrite NPs, (**B**) Capsaicin coated CoFe_2_O_4_ (CPCF) NPs.

SEM images of magnetic uncoated- CoFe_2_O_4_ NPs and Capsaicin coated CoFe_2_O_4_ (CPCF) NPs are shown in Fig. 5A,B. As indicated in (Fig. 5A), the synthesized CoFe_2_O_4_ NPs are spherical in shape, uniformly-aggregated and the grain size ranged between 15 and 25 nm. Also, the SEM Image of the Capsaicin coated CoFe_2_O_4_ (CPCF) NPs (Fig. 5A), showed that the coated nanoparticles are also spherical in shape and ranged between 25 and 35 nm.

These findings may serve as indirect evidence that the (capsaicin) shells are roughly 10 nm thick and the magnetic core/shell particles are single crystals with an mean diameter of 30 nm. According to the results, the (Capsaicin) layer is consistently loaded on CoFe_2_O_4_ NPs., as indicated in (Fig. 5B).

**Figure 6:**
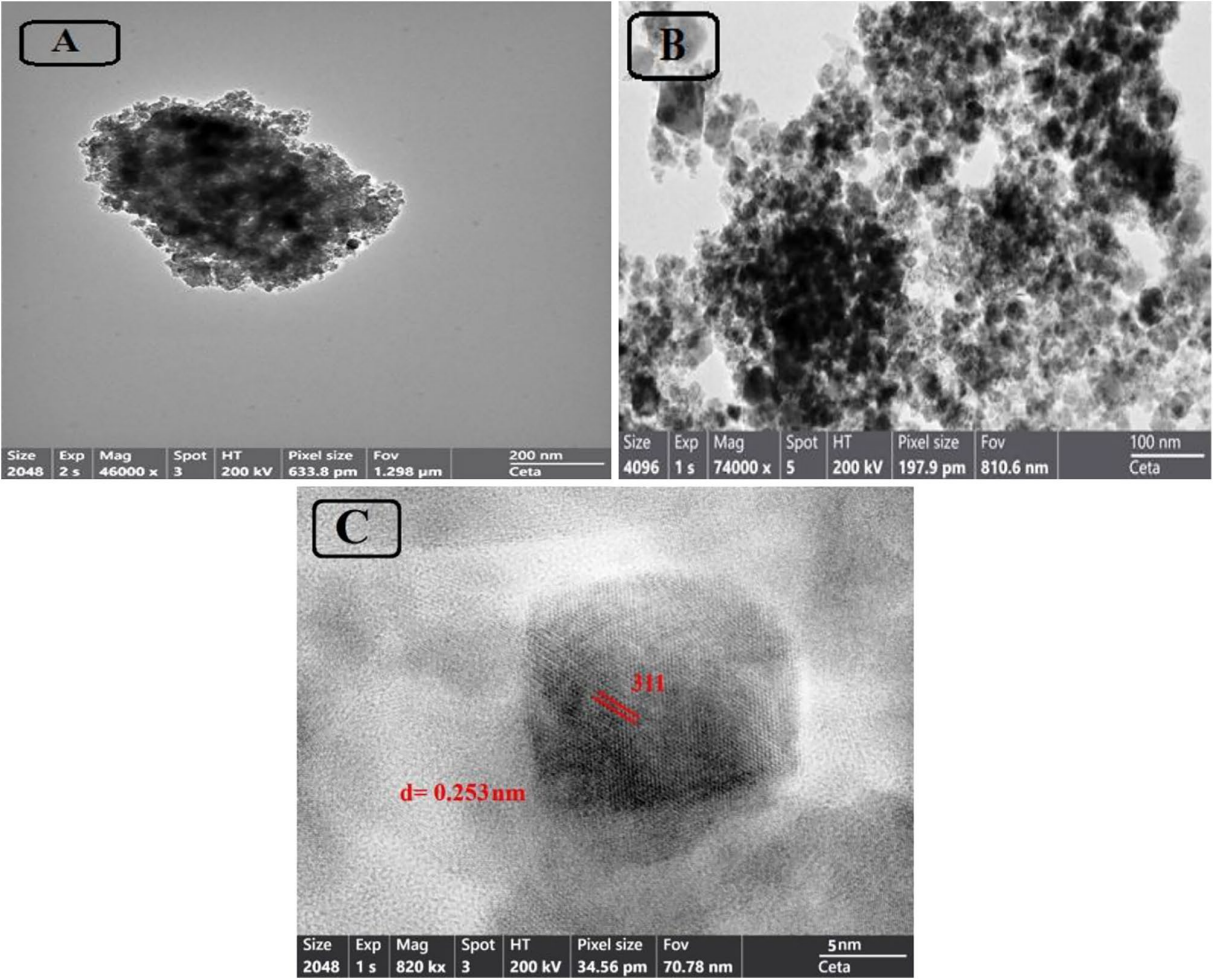
TEM images of (**A**) Nacked CoFe_2_O_4_ NPs, (**B**) Capsaicin coated CoFe_2_O_4_ (CPCF) NPs and (**C**) HRTEM image of CPCF nanocomposite with d spacing value = 0.253 nm.

TEM Images show the Shape and average particle size determination of the prepared NPs (Fig. 6A–C). The particle size and shape of naked Co Fe_2_O_4_NPs(Fig. 6A) and CPCF NPs (Fig. 6B) show that the all synthesized NPs shape are spherical and the mean particle size is found to be approximately 18.0 nm. Also, the capsaicin loaded on Co Fe_2_O_4_NPs can inhibit particle aggregation without signeficant change in particle size. The lattice fringes of as-prepared CPCF NPs can be seen obviously in Fig. 6C, the adjacent fringe spacing is about 0.253 nm, corresponding to the (311) lattice planes of Co Fe_2_O_4_NPs^47,48^. it can be seen the particles are nano sized and revealed it is in cubic shape and the average particle size of 18 nm which is agree well with the XRD result.

#### UV–Visible absorption, band gap and photoluminescence (PL) analysis for synthesized CPCF nanocomposite

**Figure 7:**
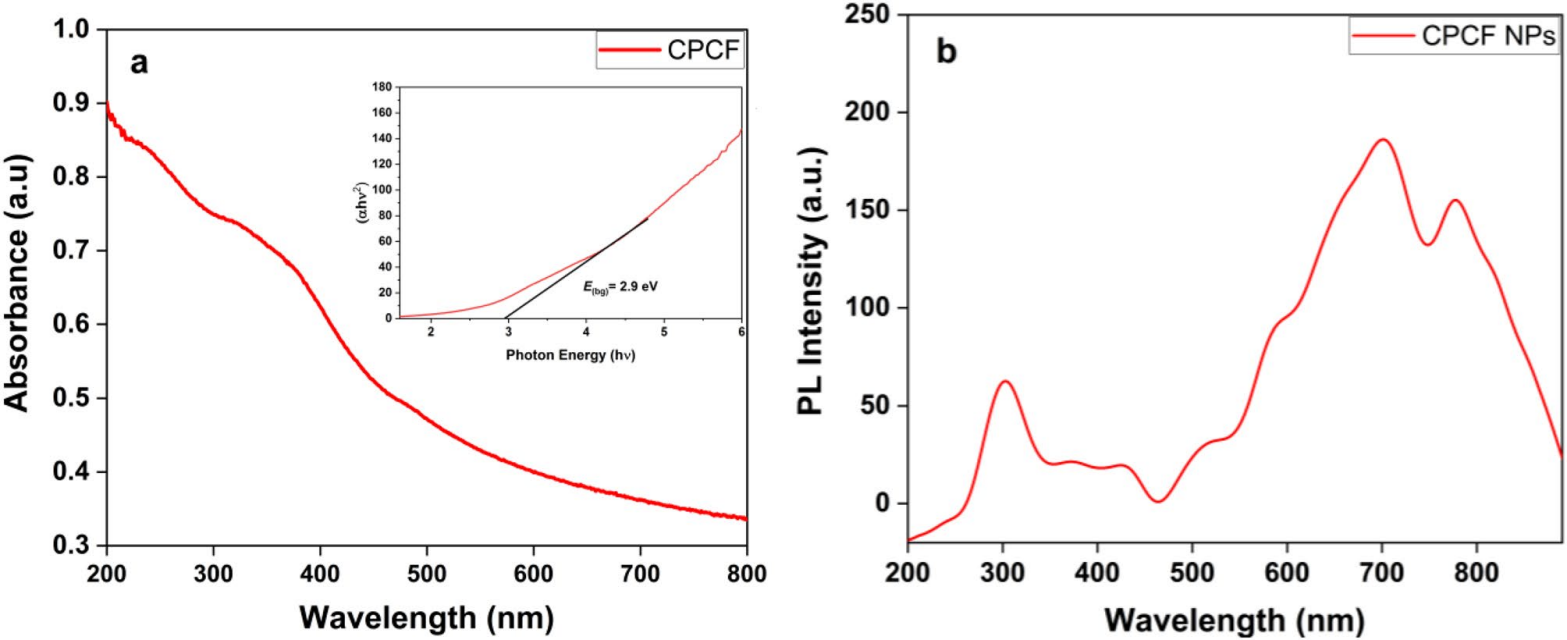
UV–Visible spectrum and band gap analysis (**a**), and Photoluminescence (PL) analysis (**b**) for synthesized CPCF nanocomposite.

Optical properties of prepared CPCF nanocomposite was analyzed in the range of 200–800 nm. Optical absorption was used to evaluate the energy gap of the nanostructures shown in Fig. 7a. As can be seen from Fig. 7a, the nanocomposite has low absorbance in the visible regions and high absorbance in the ultraviolet region^49^. the UV absorption band is observed in the region 330–500 nm, which originates primarily from the absorption and scattering of light by the CPCF nanocomposite. The band gap energy was determined from the absorption spectra using Tauc relation^50^, as shown in the inset of Fig. 7a, and found to be around 2.9 eV. It should be mentioned here that with higher band gap energy, the recombination rate of electrons and hole pairs are retarded, and photocatalytic properties are enhanced^51^**.**

Figure 7b shows the photoluminescence (PL) spectra of CPCF nanocomposite taken at an excitation wavelength of 365 nm, The photoluminescence spectrum shows two major peaks, one at 698 nm assigned to the bandgap excitons^52^ and the other at ~ 780 nm attributed to the surface-related emission (or more delicately, both surface and interface)^53^**.**

### Antimicrobial activity of the synthesized CPCF nanocomposite

**Figure 8:**
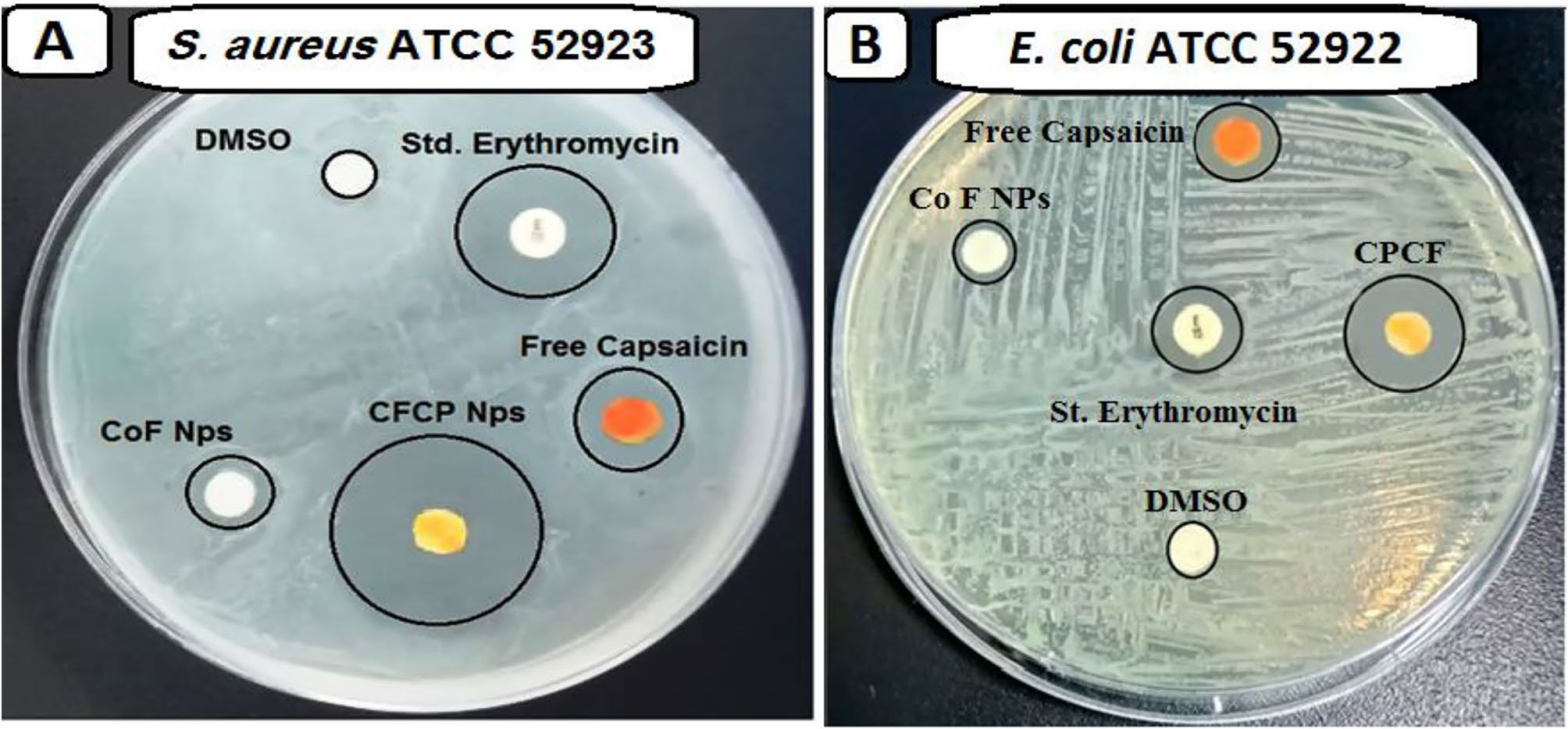
Antimicrobial activity of CoFe_2_O_4_ NPs, Free Capsaicin, and CPCF nanocomposite measured as ZOI (mm) against (**A**) *S. aureus* ATCC 52923, (**B**) *E. coli* ATCC 52922.

It is observed from the disc agar distribution method that, the synthesized CoFe_2_O_4_ and Capsaicin represented a qualitative antimicrobial potential toward the tested bacteria. According to the in-vitro ZOI result, The synthesised CPCF nanocomposite demonstrated its encouraged antibacterial activity against *S. aureus* (23.5 mm ZOI; Fig. 8A), and *E. coli* (17.0 mm ZOI; Fig. 8B) as listed in Table 2. It is worth noting that the antibacterial activity of CPCF nanocomposite was significantly higher than CoFe_2_O_4_ NPs, Free Capsaicin, and standard antibacterial agents (Erythromycin; E), which suggested the possibility of a positive synergy between Capsaicin and CoFe_2_O_4_ NPs. It's significant to suppose that the CPCF nanocomposite was more active against Gram-positive bacteria than Gram-negative bacteria. Unlike Gram-positive bacteria, which combine highly compact peptidoglycan forms, Gram-negative bacteria's cell walls are made up of layers of lipid, lipopolysaccharide, and peptidoglycan^54^.

**Table 2:** Antimicrobial activities of CoFe_2_O_4_ NPs, Free capsaicin, and CPCF nanocomposite against gram-positive and gram-negative bacteria measured as ZOI (mm) and MIC (µg/ml).

Pathogenic bacteria	ZOI of CoFe_2_O_4_ (10.0 µg/ml) (mm)	ZOI of capsaicin (10.0 µg/ml) (mm)	ZOI of CPCF (10.0 µg/ml) (mm)	MIC of CPCF NPs (µg/ml)	E
*S. aureus*	7.5	12.3	23.5	0.625	17.4
*E. coli*	9.7	10.0	17.0	1.250	11.6

The MIC results of CPCF Nanocomposite against *S. aureus* and *E. coli* were 0.625 and 1.250 µg/ml respectively as mentioned in Table 2.

### Mechanism of antimicrobial activity of the synthesized CPCF nanocomposite

**Figure 9:**
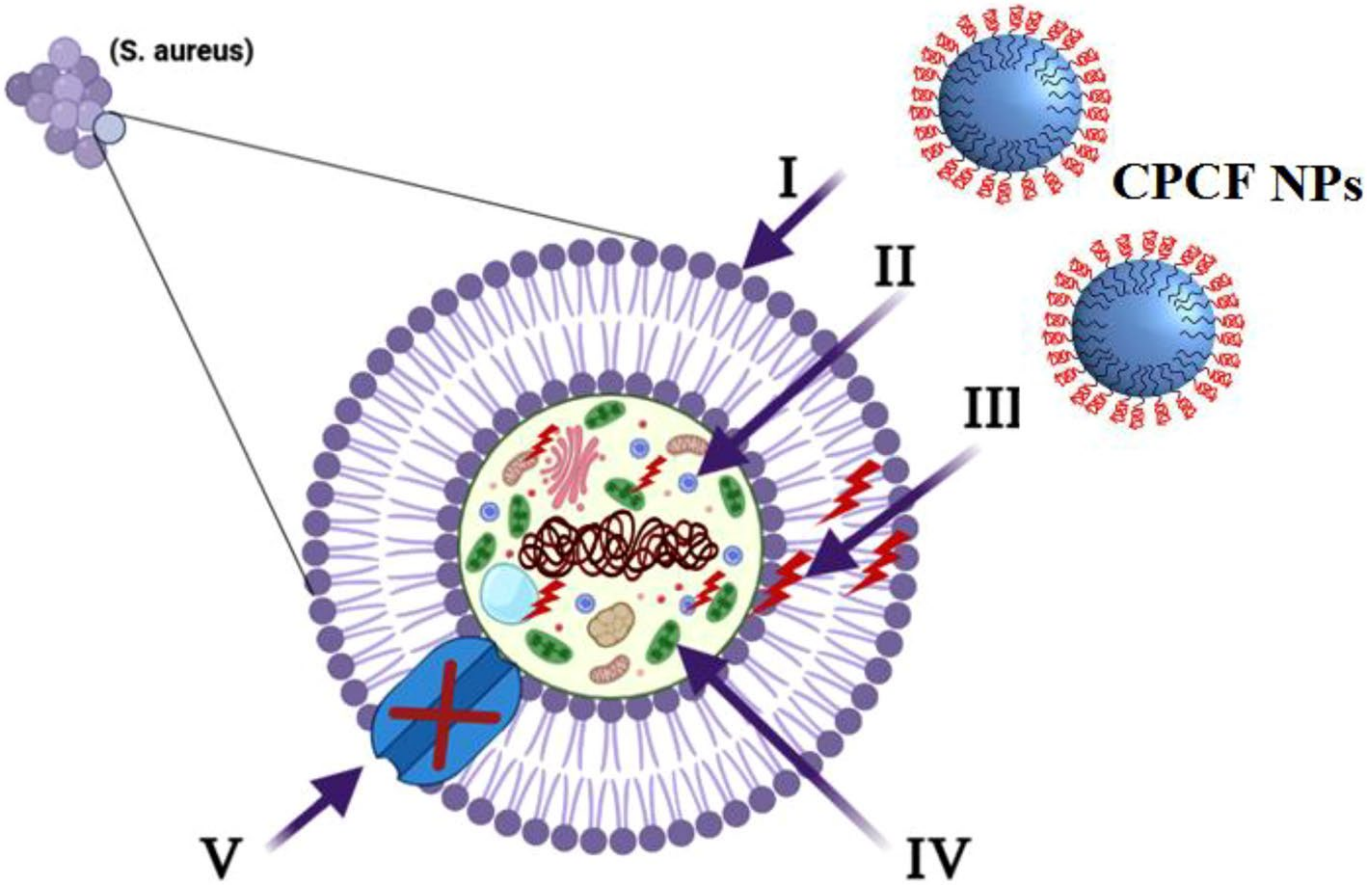
Schematic representation of the four main pathways underlying the antibacterial potential of CPCF nanocomposites: (I) the CPCF nanocomposite adhere to and wrap the microbial cell surface, resulting in the release of capsaisin, causing membrane damage and altered transport activity. (II) CPCF nanocomposite penetrate the microbial cells and interact with cellular organelles and biomolecules (such as plasmid DNA, ribosomes, chromosomal DNA, and mesosomes), affecting the respective cellular machinery. (III) CPCF nanocomposite creates and increases ROS, leading to cell damage. (IV) CPCF nanocomposite modulate the cellular signal system and causing cell death. (V) Finally, CFCP nanocomposite blocks the ion transport from and to the microbial cells.

The proposed antibacterial mechanism is depicted schematically in Fig. 9. First, the CPCF nanocomposites wrap around and adhere to the microbial cells' outer surface, rupturing their membranes and altering their transport capacity^55^. Then, all internal components, including plasmid, DNA, and other crucial organelles, are divided by the dispersion of the capsaicin-coated cobalt ferrite nanoparticles inside the microbial cell. Ultimately, Cellular toxicity ultimately results from the oxidative stress brought on by the production of ROS. Finally, nano-composite prevent the transfer of ions to and from microbial cells^56^.

### Photocatalytic degradation of fuchsine basic (FB) using magnetite NPs coated with capsaicin

**Figure 10:**
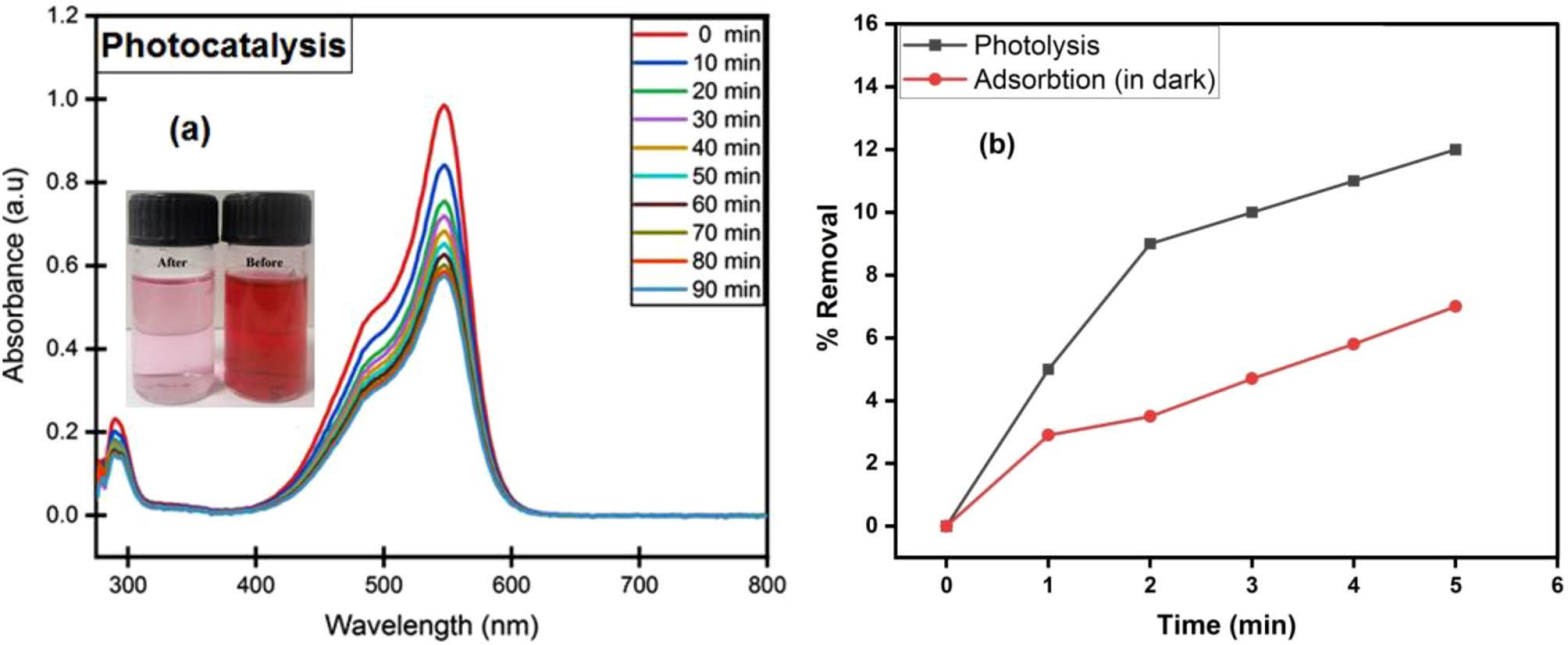
Absorbance reduction of FB with time due to: (**a**) photocatalysis (using CPCF), (**b**) % removal of Photolysis (without CPCF), and Adsorption activity of CPCF (In Dark).

At the max wavelength of 546 nm, the FB removal was measured spectrophotometrically^57^. It can be observed from Fig. 10a, as the UV irradiation time increased, it was found that the absorption peaks gradually decreased as a result of photodegradation of FB by the CPCF photocatalyst. The dye degradation percentage based on the intensity of pure FB dye at 546 nm before and after photocatalytic treatment with CPCF nanoparticles was measured to be 76.8. These results indicate that the higher surface to volume ratio of CPCF nanoparticles helps to accommodate a higher degree of dye molecular adsorption on their surface, and leads to degradation upon UV light excitation. Figure 10b shows that the degradation of FB due to photolysis after 5 h was only 12.0%, while the removal due to adsorption in the dark was around 7.0% after the same amount of time, as shown in Fig. 10b. The photograph shown in the inset of Fig. 10a was taken right after the photocatalytic reaction after 90 min, and the larger difference in the color of the dye solution between before and after photocatalytic treatment can be seen, which proved that CPCF nanomaterials are efficient photo-catalysts towards organic dyes.

#### Effect of pH on removal of FB

**Figure 11:**
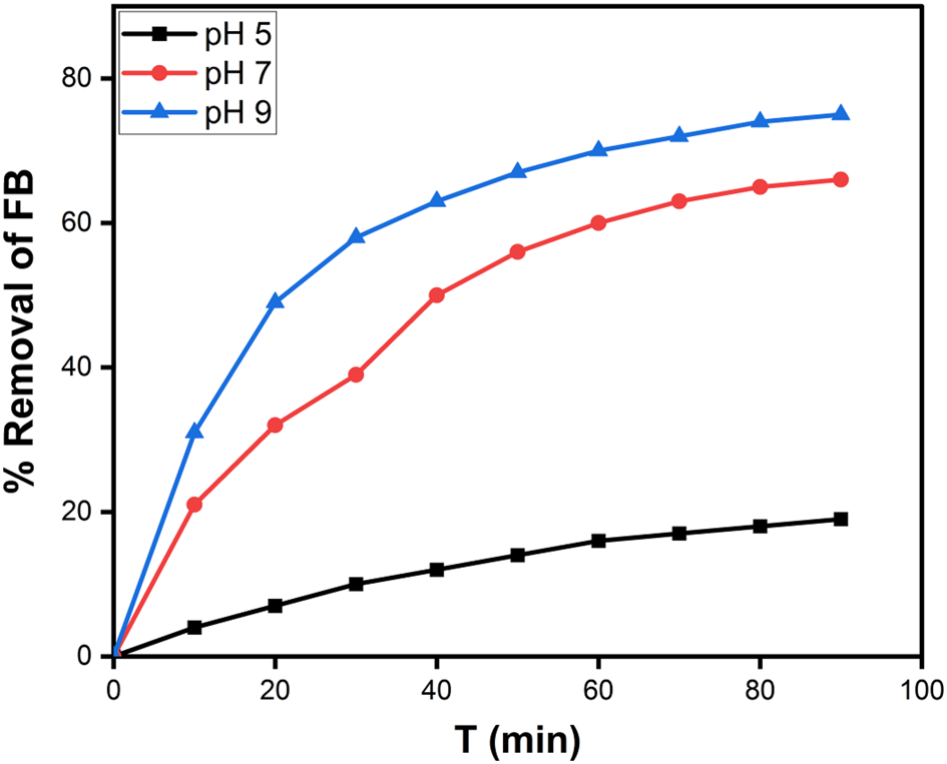
Showing the variation of FB removal (%) with time at different solution pH (5.0, 7.0 and 9.0) (10 mg g of CPCF in 50 ml of 10 mg/l FB at 25 °C).

One of the most important aspects of photocatalysis research is its sensitivity to solution pH. The influence of starting FB solution pH values was evaluated for 90 min under specified experimental conditions (10 mg of the prepared nanocomposite, 50 ml of 10 mg/L FB solution, Temp., = 25 °C). FB removal activity with time at different solution pH (5.0, 7.0, and 9.0) is represented in Fig. 11. The highest FB Removal % was recorded at pH 5.0. 0.01 g (CPCF NPs) was added to 50 mL to determine the point of zero charges (PZC) of the CPCF nanocomposite (0.01 M NaCl solution). The pH of the solutions was adjusted to 2, 4, 6, 8, 10, and 12 using HCl or NaOH. For 48 h, the samples were agitated at 200 rpm. After magnetic separation, the pH values of the solutions were determined (CPCF NPs).

**Figure 12:**
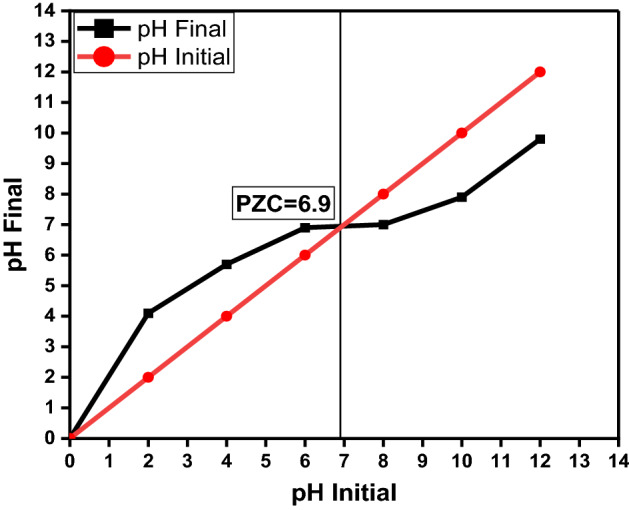
Point of zero charges (PZC) of CPCF at different pH.

The PZC value was calculated by plotting the final pH vs the initial pH. Figure 12 exhibits these findings. According to Fig. 12, the PZC was found to be at pH 6.9 when there was no significant change between the final and initial pH values. It indicates that when pH < PZC and pH > PZC, the surface charge of the photocatalyst (CPCF NPs) is positive and negative, respectively. Furthermore, when the pH of the solution equals the pH of the PZC, the surface charge of the photocatalyst is neutral, and the electrostatic interaction between the photocatalyst surface and ions (FB ions) is negligible^58^. As a result, the positive charge of FB is now attracted to the negative charge on the surface of the CPCF NPs photocatalyst, which enhances the degradation of FB. At pH 5.0, the degradation of FB dropped. This happens because the net surface charge of the CPCF NPs is positive at this point and there are repulsive forces between the two positive charges of the FB and the CPCF nanocomposite.

#### Effect of initial FB concentration

**Figure 13:**
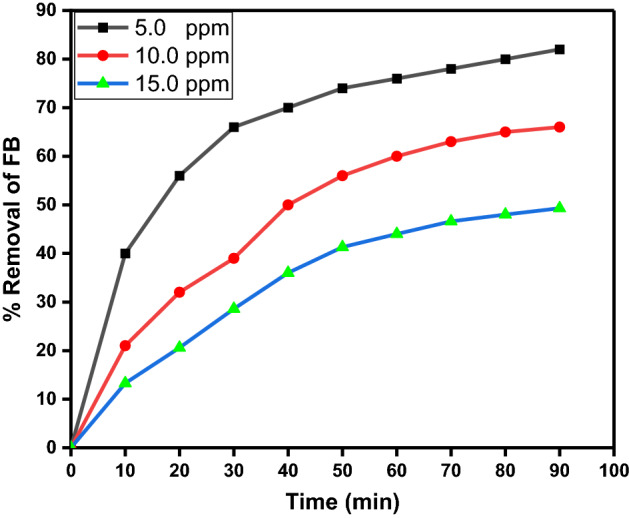
The variation of percent removal as a function of contact time at different initial FB concentrations (10, 20, and 30 mg/l) at pH 9.0 and 10.0 mg CPCF.

Because the initial FB concentration is so vital in the removal process, the influence of FB ionic strength was examined by altering the initial FB concentration while remaining the other reaction conditions unchanged. Figure 13 illustrates the change in removal percentage as a function of contact time for different initial FB concentrations (5.0, 10.0, and 15.0 mg/l). In accordance with the results, the degradation efficiency is inversely proportional to the concentration of FB, which may be successfully removed in the presence of the synthesized CPCF nanoccatalyst under UV light irradiation even at high initial concentrations.

#### Effect of CPCF nanocatalyst dosage on photodegradation efficiency

**Figure 14:**
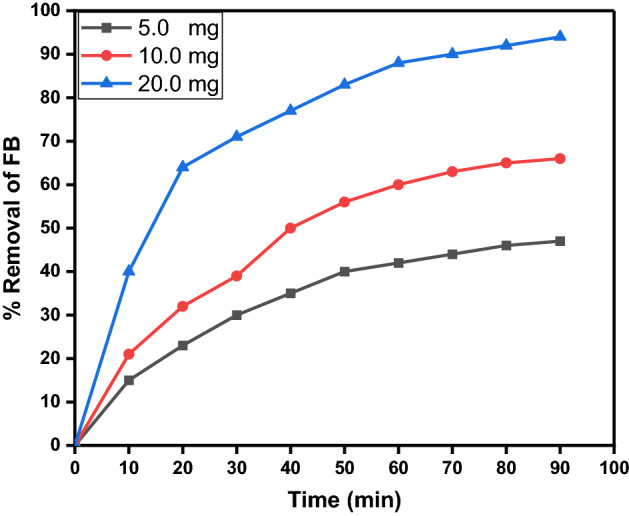
Effect of the photocatalyst dose on the Removal efficency of FB (50 ml FB solution (10 mg/l), Temp. = 25 °C and pH 9).

For studying the effect of CPCF nanocatalyst dosage on the removal behavior of FB under UV light, the amount of photocatalyst was altered from 5 to 20 mg without any change in other parameters, as shown in Fig. 14. The results showed the photodegradation efficacy was increased with increasing the CPCF photocatalyst amount. This direct proportional relationship might be due to an increase in surface area of CPCF photocatalyst to volume ratio of FB ions in the reaction solution^59^. Also, the particle size of a photocatalyst is one of the factors that determine the photon utilization efficiency. Many reports have confirmed the significant effects of particle size on photocatalytic activity^60,61^. It is generally considered that the grain size of a photocatalyst should be small; i.e., the specific surface area should be large. If the grain size is small, the transport of photogenerated electrons (e^−^) and holes (h^+^) from the bulk to the surface becomes easier^62^. Moreover, the surface charge transfer rate will be improved by an increase in the amount of reactant adsorption. The photoabsorption properties of semiconductors also depend on the particle size in the nanometer range^63^.

Based on XRD, TEM, and SEM analyses, it is found that the synthesized CPCF nanoparticles shapes are spherical and the mean particle size is found to be approximately 18.0 nm. Accordingly, CPCF nanoparticles have a large surface area and a broadened band gap, furthermore, they contain more active sites and display improved photocatalytic activity.

### Kinetic studies

**Figure 15:**
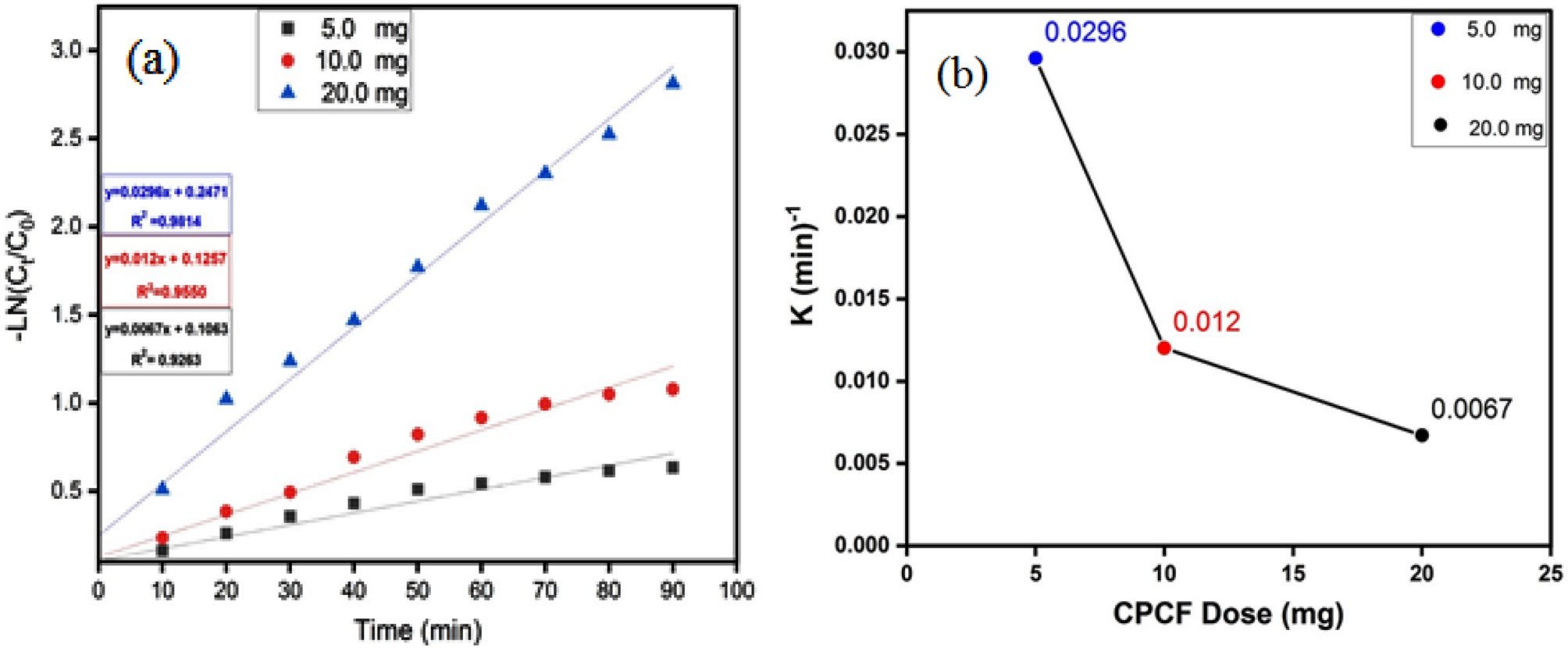
(**a**) Kinetics plots for linear fitting of data obtained from pseudo-first-order reaction model for FB degradation under UV light irradiation and initial concentration 10 ppm of FB, 50 mL of 5, 10, and 15 mg of catalyst dose and (**b**) Shows a relation of apparent pseudo-first-order rate constants vs. initial concentration of FB.

The rate of FB degradation can be determined using the following equation:

5 $$- ln^{Ct} /_{CO} = \, - Kt$$

where, t is the removal time, k is the removal rate constant, and (Ct and C)_o_ are the corresponding initial and remaining concentrations of FB. Figure 15. represent a relation of (− ln Ct/Co ) vs. t

The results indicated that the kinetics of the removal process followed pseudo-first-order rate laws. Furthermore, as shown in Fig. 15b, an increase in catalyst dosage results in a decrease in the apparent pseudo-first-order rate constants. This reliance on reaction rate constants on FB concentration is well with the presented literature^64,65^.

#### Mechanism of photocatalysis of FB

As mentioned in many studies of literature, the possible mechanism is as follows^66,67^. Changing the pH affects photodegradation methods such as hydroxyl radical attack, explicit oxidation by positive holes in the valence band, and explicit reduction by electrons in the conduction band. It is expected that photocatalytic degradation will occur in the presence of a CPCF photocatalyst because of the generation of electron–hole pairs on the surface of the used photocatalyst due to UV-irradiation. The holes' oxidative potential either interacts with the-OH groups to create hydroxyl radicals or oxidises the reactive FB to form a degradation product^57^. The reactions of FB and the used photocatalyst are given below. (Eqs. 6–9).

6 $${\text{CPCF NPs }} + {\text{ hV}} \to {\text{CPCF NPs }}\left( {{\text{e}}^{ - }_{{{\text{CB}}}} + {\text{ h}}^{ + }_{{{\text{VB}}}} } \right)$$

7 $${\text{h}}^{ + }_{{{\text{VB}}}} + {\text{CPCF NPs}} \to {\text{CPCF NPs}}^{ + } \left( {\text{Oxidation of the compound}} \right)$$

or

8 $${\text{h}}^{ + }_{{{\text{VB}}}} + {\text{ OH}}^{-} \to {\text{OH}}^{.}$$

9 $${\text{OH}}^{ \cdot } + {\text{ FB dye}} \to \left( {\text{Degradation products}} \right)$$

**Figure 16:**
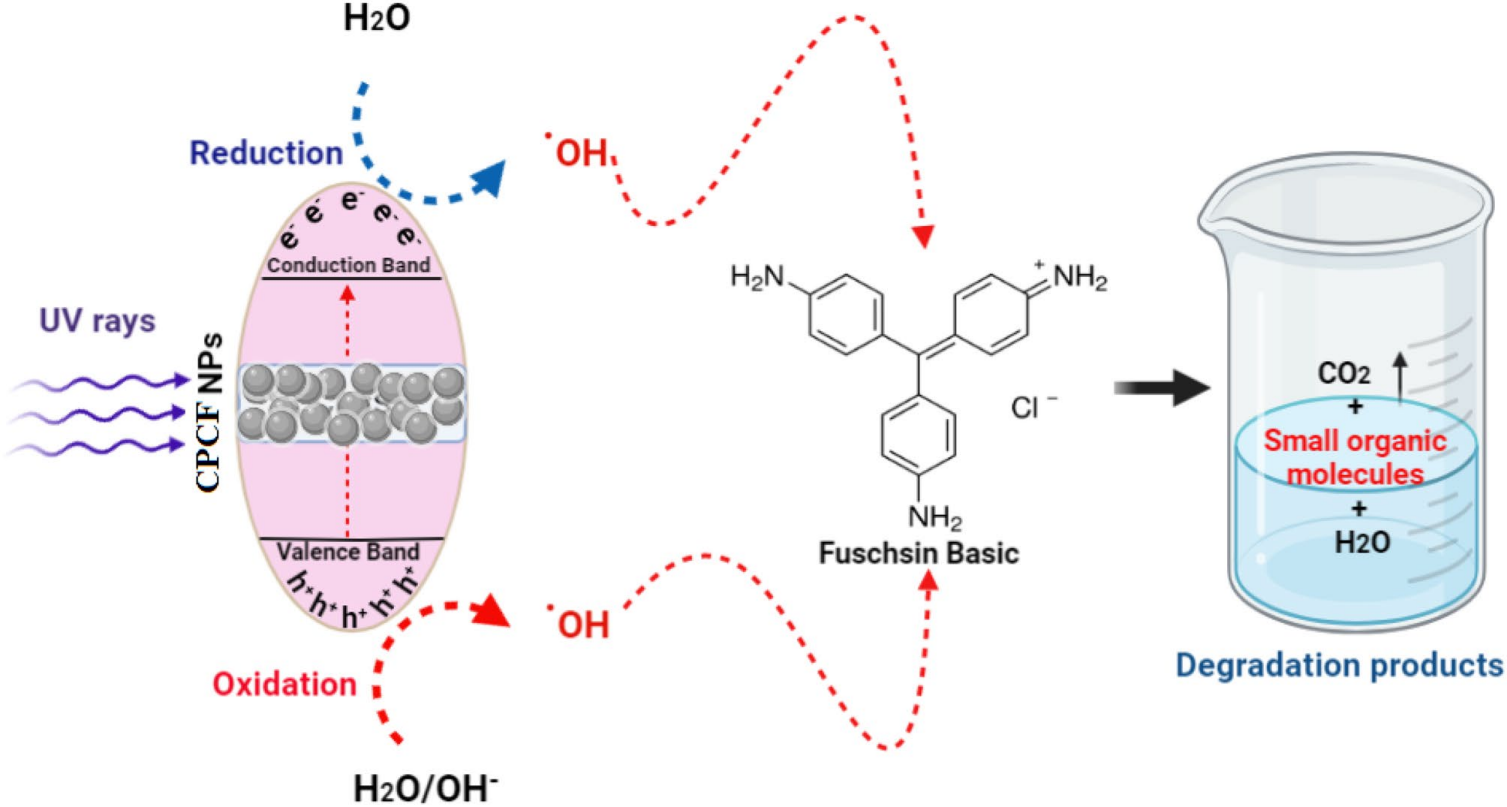
The possible photocatalytic reaction mechanism for Fuchsine basic (FB) photodegradation via CPCF nanocomposite.

Figure 16 illustrates the suggested mechanism of interaction between the produced nanocomposite and FB. The redox reactions will start once UV light has excited the CPCF NPs. The produced free radicals (such as OH^**·**^ and O_2_**·**^−^) will then decompose FB into minor organic compounds. Since there are currently no publications concerning the degradation of FB that have been reported, more studies using gas chromatography-mass spectrometry (GC–MS) and high-performance liquid chromatography (HPLC) are needed to more clearly study the degradation products of FB.

## Conclusion

CoFe_2_O_4_ NPs have been synthesized by a chemical co-precipitation method and characterized by structural and optical tools. The surface of CoFe_2_O_4_ NPs was coated with capsaicin (CAPS) by a direct addition method to obtain a modified CAPS-CoFe_2_O_4_ (CPCF) nanocomposite. The photocatalytic efficiency of the prepared (CPCF) nanocomposite was tested against Fuchsine basic (FB). Also, various parameters affecting the efficiency of removal potential such as (pH on degradation of FB, FB initial concentration, and photocatalyst dose) have been studied. Based on XRD, TEM, and SEM analyses, it is found that CoFe_2_O_3_ nanoparticles are located at the core, while the CAPS are coated in this core, producing CAPS-functionalized CoFe_2_O_4_ NPs with particle sizes varying in from 15.0 to 25.0 nm with average particle size at 18 nm. From FTIR results, the presence of strong peaks of CoFe_2_O_4_ NPs and CPCF NPs at 658 cm^−1^ was attributed to the stretching vibration mode associated with the Fe–O bond in the crystalline lattice of CoFe_2_O_4_ NPs. Moreover, the band at 515 cm^−1^ was confirmed as the metal oxide bond (Co–O) in the nanoparticle structure. Results obtained from the photodegradation of FB indicated that the maximum FB removal achieving 94.6% in equilibrium was observed using 20.0 mg of CPCF at pH 9.0. Furthermore, their antimicrobial behavior has been examined against Gram-positive (*S. aureus*) and gram-negative (*E. coli*). The in-vitro ZOI and MIC results verified that CPCF NPs are also active upon Gram-Positive *S. aureus* (23.0 mm ZOI and 0.625 ug/ml MIC) than Gram-Negative *E. coli* (17.0 mm ZOI and 1.250 ug/ml MIC). The synthesized CPCF NPs are promising for potential applications in pharmaceutical uses and wastewater treatment.

## Data Availability

All data generated or analysed during this study are included in this published article.
